# The Clinical Significance of Procalcitonin Elevation in Patients over 75 Years Old Admitted for COVID-19 Pneumonia

**DOI:** 10.1155/2021/5593806

**Published:** 2021-06-28

**Authors:** Andrea Ticinesi, Antonio Nouvenne, Beatrice Prati, Loredana Guida, Alberto Parise, Nicoletta Cerundolo, Chiara Bonaguri, Rosalia Aloe, Angela Guerra, Tiziana Meschi

**Affiliations:** ^1^Geriatric-Rehabilitation Department, Azienda Ospedaliero-Universitaria di Parma, Parma, Italy; ^2^Clinical Chemistry and Hematology Laboratory, Diagnostic Department, Azienda Ospedaliero-Universitaria di Parma, Parma, Italy; ^3^Department of Medicine and Surgery, University of Parma, Parma, Italy

## Abstract

**Aim:**

To investigate the clinical significance of procalcitonin (PCT) elevation on hospital admission for coronavirus disease-19 (COVID-19) and its association with mortality in oldest old patients (age > 75 years).

**Methods:**

The clinical records of 1074 patients with chest high-resolution computed-tomography (HRCT) positive for interstitial pneumonia and symptoms compatible for COVID-19, hospitalized in medical wards during the first pandemic wave in a single academic center in Northern Italy, were retrospectively analyzed. All patients had serum PCT testing performed within six hours from admission. Information on COVID-19-related symptoms, comorbidities, drugs, autonomy in daily activities, respiratory exchanges, other routine lab tests, and outcomes were collected. Clinical characteristics were compared across different admission PCT levels and ages. The association of admission PCT with mortality was tested separately in participants aged > 75 and ≤75 years old by stepwise multivariate Cox regression model with forward selection.

**Results:**

With increasing classes of PCT levels (<0.05, 0.05-0.49, 0.5-1.99, and ≥2 ng/ml), there was a significant trend (*P* < 0.0001) towards older age, male gender, wider extension of lung involvement on HRCT, worse respiratory exchanges, and several other laboratory abnormalities. Each incremental PCT class was associated with increased risk of hospital death at multivariate models in subjects older than 75 (hazard ratio for PCT ≥ 2 vs. <0.05 ng/ml: 30.629, 95% confidence interval 4.176-224.645, *P* = 0.001), but not in subjects aged 75 or younger.

**Conclusions:**

In patients admitted for COVID-19, PCT elevation was associated with several clinical, radiological, and laboratory characteristics of disease severity. However, PCT elevation was strongly associated with hospital mortality only in oldest old subjects (age > 75).

## 1. Introduction

Serum procalcitonin (PCT) elevation is frequently seen in patients hospitalized for moderate or severe forms of coronavirus disease-19 (COVID-19) [1]. The main trigger for PCT synthesis is the presence of circulating endotoxins [1, 2]. For this reason, in clinical practice, PCT is generally considered a biomarker of systemic bacterial infections and has been interpreted as an indicator of secondary bacterial infection in COVID-19 [2].

However, PCT synthesis can also be stimulated by the elevation of proinflammatory cytokines, including interleukin-6 (IL-6), interleukin-1b (IL-1b), and tumor necrosis factor-*α* (TNF-*α*) [1]. These mediators are massively involved in the so-called cytokine storm, typical of the progression from the viremic to the hyperinflammatory stage of COVID-19 and characterized by the onset of respiratory symptoms and interstitial pulmonary infiltrates on chest radiology [3]. Thus, PCT elevation may represent a direct consequence of the COVID-19 cytokine storm and could also be interpreted in the framework of a “viral sepsis” syndrome [4].

Several systematic reviews and meta-analyses have indicated PCT elevation as a prognostic biomarker of adverse outcomes in COVID-19, including progression from moderate to severe and critical forms of the disease, intensive care unit (ICU) admission, need for mechanical ventilation, and mortality [5–15]. PCT shares this prognostic role with many other biomarkers of inflammation, including IL-6, C-reactive protein (CRP), D-dimer, and white blood cell (WBC) elevation [5–10].

However, most of the evidence linking PCT elevation on hospital admission for COVID-19 with adverse outcomes is based on small-sized studies with retrospective design, conducted on Chinese populations during the very early phases of the COVID-19 pandemic [6–10]. The demography of such populations is very different from that of Western patients admitted for COVID-19 that were generally older and with a higher burden of multimorbidity and frailty [16]. Data on the prognostic role of PCT elevation in these age groups are scarce.

In older subjects, inflammaging, immune senescence, and chronic inflammatory comorbidities can represent triggers to PCT synthesis even in the absence of infection, limiting the diagnostic and prognostic role of this biomarker [17–19]. Studies conducted in geriatric patients with bacterial infection, however, suggest that PCT maintains a prognostic role in these age-groups, though with different cut-offs than in younger subjects [20–22].

The aim of this retrospective study was to investigate the association of PCT elevation on hospital admission for COVID-19 with mortality in patients older and younger than 75 years old, to clarify the prognostic significance of this biomarker in geriatric patients.

## 2. Methods

### 2.1. Study Setting and Population

The study was conducted at the Geriatric-Rehabilitation Department of Parma University-Hospital in Northern Italy during the first pandemic wave (Feb 28-Jun 10, 2020). This department was the main hub for the care of patients with COVID-19 not requiring intensive support of vital functions of an area of >300,000 inhabitants, including the urban area of Parma and several neighboring villages. The department has a capacity of 195 medical beds that were extended up to 330 in periods of extreme overflow of patients needing urgent care for COVID-19 [23].

Patients were admitted after an Emergency Department (ED) visit for typical symptoms of COVID-19 and after undergoing a high-resolution chest computed tomography (HRCT), with results compatible with the presence of interstitial pneumonia of viral origin [23]. Nasopharyngeal swab for the detection of Severe Acute Respiratory Syndrome CoronaVirus-2 (SARS-CoV-2) with Reverse-Transcriptase Polymerase-Chain Reaction (RT-PCR) was performed immediately after admission, as diagnostic confirmation [23]. These criteria of admission were based on the assumption that, during a pandemic wave, clinical and radiological findings typical of COVID-19 must be attributed to the disease with reasonable certainty even in the absence of an immediate molecular diagnostic confirmation. In fact, nasopharyngeal swabs frequently tested negative for SARS-CoV-2 on hospital admission during the first pandemic wave, though patients were confirmed as having COVID-19 at a later stage [24].

The total number of patients admitted during the first pandemic wave (Feb 28-Jun 10, 2020) were 1684. Included in this study were 1074 patients with interstitial pneumonia confirmed on HRCT performed on the day of admission and with serum PCT levels tested within six hours from ward admission. We excluded all subjects under 18 years old, those lacking blood tests within six hours from ward admission, those with known conditions inducing severe immune suppression and those with indeterminate findings on chest HRCT.

The patients, who tested negative for SARS-CoV-2 on the day of admission, but exhibited typical radiologic signs of viral pneumonia, clinical presentation compatible with COVID-19, and a history of contact with confirmed COVID-19 cases were not excluded from the study, assuming false-negative molecular testing that was reported as very common in the early phases of the pandemic [25].

### 2.2. Data Collection and Outcome Measures

We retrospectively reviewed the clinical records of eligible patients, collecting demographic, clinical, and laboratory data. Namely, age, gender, duration, and type of symptoms before admission, number of chronic comorbidities, number of drugs, and dependency in daily activities were retrieved from each patient's history. Treatment with angiotensin-converting enzyme inhibitors (ACE-I) or angiotensin receptor blockers (ARBs) was particularly considered for its possible association with COVID-19 outcome. The characteristics of chest HRCT findings, including presence of consolidations and extension of ground-glass opacity abnormalities, determined using a visual scoring system described elsewhere [26], were also recorded from each clinical record. Abnormalities on the electrocardiogram (ECG) were also classified for each participant.

The results of RT-PCR testing on nasopharyngeal swabs for detection of SARS-CoV-2 performed within 12 hours from admission were collected.

Routine blood tests performed within 6 hours from ward admission included PCT, CRP, complete blood cell count, creatinine, urea, sodium, potassium, total bilirubin, aspartate aminotransferase (AST), creatine-phosphokinase (CPK), lactate dehydrogenase (LDH), fibrinogen, and D-dimer. Serum IL-6 levels were also determined in selected cases. The serum PCT levels were tested by a paramagnetic particle chemiluminescent immunoassay named Access PCT (Beckman Coulter, Brea, CA, USA).

Respiratory exchanges were evaluated in all participants on admission through the calculation of PaO_2_/FiO_2_ (arterial pressure of oxygen divided per the fractional oxygen flow administered).

The clinical course of COVID-19 was evaluated in this study through collection of the worst value of PaO_2_ at blood gas analysis and the highest oxygen flows administered.

In-hospital mortality was considered as the main outcome, while need for noninvasive ventilation (NIV) and ICU transferal were considered as secondary outcomes.

### 2.3. Statistical Analysis

Variables were expressed as median and interquartile range (IQR) or as percentages, as appropriate. The normality of distribution of continuous variables was assessed with the Kolmogorov-Smirnov test. PCT levels on admission were considered as the independent variable. The population was divided into four groups, according to PCT levels: <0.05 ng/ml (group 1, normal), ≥0.05 ng/ml and <0.5 ng/ml (group 2, mild elevation), ≥0.5 ng/ml and <2 ng/ml (group 3, severe elevation), and ≥2 ng/ml (group 4, extreme elevation). These cut-offs were compatible with those used for prescription of antibiotic therapy in suspected bacterial infections [27]. All demographic, clinical, laboratory, and outcome data were compared across these four groups using Kruskal-Wallis test, linear regression, univariate general linear model, one-way analysis of variance (ANOVA) for continuous variables, and Pearson chi-square test and logistic regression for dichotomous variables. All demographic, radiological, and virological characteristics showing a significantly different trend across PCT classes were considered as potential confounders in regression models. In particular, age, sex, positive RT-PCR test for SARS-CoV-2, and presence of consolidations on chest HRCT were considered as possible confounders, for their established influence on COVID-19 clinical presentation and prognosis [28, 29].

Then, the association of different PCT classes upon admission with in-hospital mortality was evaluated separately for patients older than 75 and for patients aged 75 or younger with Cox regression multivariate analysis. Included as possible confounders were all demographic, radiological, and virological characteristics with significantly different trend across PCT classes (namely, age, sex, presence of consolidations on chest HRCT, and positive nasopharyngeal swab for SARS-CoV-2) and treatment with ACE-I or ARBs, for its possible association with COVID-19 outcome.

*P* values were considered significant when <0.05. Statistical analyses were performed with the SPSS package (v.26, IBM, Armonk, United States).

### 2.4. Ethical Statement

The study was approved by the local Ethics Committee as part of a larger retrospective project on the clinical correlates of COVID-19 in hospitalized patients (ID 273/2020/OSS/AOUPR, Comitato Etico dell'Area Vasta Emilia Nord). Due to the retrospective design of the study, informed consent was obtained only whenever possible, in compliance with the current regulations.

## 3. Results

Among the 1074 patients included in the study, 524 were older than 75 years old, while 550 were aged 75 or younger. Only 130 patients had normal serum PCT values on admission (<0.05 ng/ml, group 1), while the numbers of patients classified as having mild elevation (group 2), severe elevation (group 3), and extreme elevation (group 4) were 698, 145, and 101, respectively (Table 1). There was a significant trend of older age (*P* < 0.001), reduced representation of female gender (*P* < 0.001), wider extension of lung parenchyma abnormalities on chest HRCT (*P* < 0.001), and higher prevalence of nasopharyngeal swabs positive for SARS-CoV-2 (*P* = 0.032) with increasing classes of PCT level (Table 1).

PCT elevation on admission was also positively associated with dependency in daily activities, number of comorbidities, number of drugs, and presence of obesity and chronic kidney disease (Table 2). The chronic use of medications acting on the renin-angiotensin-aldosterone system, such as angiotensin-converting enzyme inhibitors or angiotensin receptor blockers, was not different across PCT classes (Table 2). Patients with extreme PCT elevation had also a shorter duration of COVID-19-related symptoms before admission and higher prevalence of dyspnea and electrocardiogram abnormalities (Table 2).

A trend towards worse respiratory exchanges was also identified with increasing PCT levels (PaO_2_/FiO_2_ group 1: 357 mmHg, IQR 290-406; group 2: 257 mmHg, IQR 173-333; group 3: 141 mmHg, IQR 84-260; group 4: 171 mmHg, IQR 87-278; *P* < 0.001 adjusted for age, sex, consolidations, and positive nasopharyngeal swab) (Table 3). PCT elevation was also associated with a higher prevalence of laboratory abnormalities on admission, namely, neutrophilia, lymphopenia, elevation of D-dimer, fibrinogen, AST, LDH, and CPK (Table 3 and Table 4). It was also associated with an increasing trend of elevation of other inflammatory markers, including CRP and IL-6 (Table 3).

As shown in Table 4, classes of PCT elevation on admission were associated with increased frequency of noninvasive ventilation, ICU admission, and hospital mortality. Higher rates of mortality with increasing admission PCT classes were observed in both patients aged ≤ 75 (group 1: 3%; group 2: 10%; group 3: 17%; group 4: 25%) and in patients older than 75 (group 1: 5%; group 2: 33%; group 3: 55%; group 4: 68%), but mortality was significantly higher for each PCT class in subjects older than 75 (Table 4).

**Figure 1:**
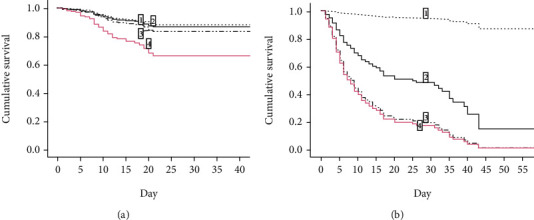
Survival functions, determined by Cox regression analysis, of participants stratified according to levels of procalcitonin on admission (level 1: <0.05 ng/ml; level 2: ≥0.05 and <0.5 ng/ml; level 3: ≥0.5 and <2 ng/ml; level 4: ≥2 ng/ml) and age ((a) age ≤ 75 years old; (b) age > 75 years old).

In multivariate Cox regression analysis models, admission PCT classes discriminated the risk of mortality (*P* < 0.001) only in patients older than 75 years old (Table 5, model 2; Figure 1(b)) and not in patients aged 75 or younger (Table 5, model 1; Figure 1(a)), where age was instead the main factor associated with hospital mortality (HR 1.134, 95% CI 1.082-1.189, *P* < 0.001).

## 4. Discussion

In a large group of patients admitted with clinical and radiological findings compatible with COVID-19 pneumonia during the first pandemic wave in an academic hospital in Northern Italy, admission levels of serum PCT were associated with more severe characteristics of disease. However, PCT was significantly associated with the risk of adverse outcomes only in subjects older than 75 years old. In patients aged 75 and younger, instead, PCT was not so good in discriminating the risk of mortality, especially for levels <2 ng/ml.

These results confirm the role of PCT as a possible biomarker of severe COVID-19 and predictor of adverse outcomes. However, in comparison with previous research, they contribute to clarify in which patient category PCT testing is associated with more prognostic information. In fact, PCT testing on admission for COVID-19 may be of particular importance in geriatric patients, where this biomarker is not only an indicator of severe disease but also a prognostic biomarker.

The diagnostic and prognostic value of PCT elevation in geriatric patients on hospital admission has been investigated by several studies in the pre-COVID-19 era, with conflicting results [17–22, 30–34]. Some of these studies have concluded that PCT elevation in older patients is significantly associated with bacterial pneumonia, sepsis, severity of infection, and mortality [20–22, 30, 31]. Other studies, instead, underlined that the diagnostic and prognostic performance of PCT elevation in these patients is nonsuperior and in some cases deliberately inferior, to that of serum CRP [19, 32–34], which is generally considered as the inflammatory biomarker guiding therapeutic choices in acute geriatric patients [35, 36]. Therefore, PCT elevation was basically regarded as a marker of inflammation or infection in older patients and not as a reliable diagnostic or prognostic indicator [34].

In comparison with other pulmonary infections, severe COVID-19 is characterized by massive activation of the inflammatory response, which is substantiated in the so-called “cytokine storm” with elevation of IL-6 and TNF-*α* in blood. PCT elevation could represent a consequence of this pathological phenomenon characterizing the transition between the viremic and the organ damage phases of COVID-19, although it could also indicate some degree of bacterial superinfection on pulmonary interstitial lesions [3, 37].

In older patients, preexisting frailty and multimorbidity could be associated with chronic activation of the inflammatory system, the so-called inflammaging [38, 39]. We can hypothesize that this condition exacerbates the reactivity of the inflammatory and immune system to SARS-CoV-2 infection, promoting the cytokine storm and resulting in increased levels of inflammatory biomarkers, including PCT, in comparison with adult subjects. Recent studies have in fact suggested that the presence of frailty and multimorbidity could represent risk factors for adverse outcomes of COVID-19 beyond chronological age [40–42], although the severity of clinical presentation of respiratory failure remains the strongest prognostic indicator in all patients [42, 43]. The pronounced association between PCT elevation and hospital mortality, observed in participants over 75 years old, could thus represent the effect of increased burden of frailty and multimorbidity in such patients.

These conditions could also drive an increased risk of pulmonary bacterial superinfection in patients hospitalized for COVID-19. In this case, the association between PCT elevation and mortality could simply represent an indicator of this complication of COVID-19. However, bacterial superinfections are difficult to detect in COVID-19 patients, especially in a context of massive hospital overcrowding as the one in which our investigation was conducted. The few studies that were specifically focused on this issue gave conflicting results [44, 45]. For example, Falcone and colleagues found a substantial prevalence of bacterial and fungal superinfections (21.9%) in a group of 315 patients hospitalized for COVID-19 in Italy [44], while Garcia-Vidal and colleagues showed that bacterial superinfections were uncommon (prevalence 3.1%) in their cohort of 989 hospitalized patients [45]. Thus, the current evidence does not allow to attribute PCT elevation exclusively to bacterial superinfection in patients hospitalized for COVID-19.

In our study, the presence of bacterial superinfection was not systematically assessed, due to the emergency situation and overcrowding of hospital wards. Blood and sputum cultures could be obtained only in a minority of cases and were negative for the presence of pathogens. However, the circumstance that the trend of prevalence of pulmonary consolidations on chest CT, a possible sign of bacterial superinfection in interstitial pneumonia, was not significant with increasing PCT classes (Table 1) suggests that other mechanisms could be involved in PCT elevation.

Most studies that investigated the association between PCT elevation and prognosis of COVID-19 in hospitalized patients were mainly conducted in adult subjects and included only a very small number of seniors over 75 years old [6–10]. On the other side, the main studies that have investigated the clinical presentation and prognostic factors of COVID-19 in older individuals did not include data on PCT testing on admission [40, 41].

The only exception was the study by Chen and colleagues, who detected significantly lower serum PCT values on admission for COVID-19 in subjects older than 65 in comparison with adult subjects [46]. However, this study included just a small number of geriatric patients (only 55 participants older than 65) and was conducted in China during the very first phases of the COVID-19 pandemic [46]. Thus, the setting was substantially different from that of the present investigation, which was conducted on a much larger sample with a substantial proportion of older individuals.

The associations between PCT levels on admission and other different clinical, radiological, and laboratory parameters of COVID-19 confirmed that PCT elevation represents a marker of disease severity at all ages. However, in subjects aged 75 or younger, this association did not translate into substantial adverse outcome prediction. From the epidemiological point of view, this circumstance suggests that other factors, different from inflammatory parameters, may substantially influence the mortality risk in adult patients. Such factors could include chronic comorbidities, SARS-CoV-2 viral load, host genotype-related factors, and chronological age [12, 47, 48], but not chronic medications. Drugs like ACE-I and ARBs, that were associated with COVID-19 outcomes in previous studies from China [49], were not associated with mortality in our population, matching the findings of a nationwide study conducted in our country [50].

The association between PCT and mortality detected in geriatric patients suggests that younger subjects may be more resilient to the hyperinflammatory state associated with the second phase of COVID-19 than older patients, reflecting a reduced prevalence of frailty and chronic illness [16]. Chronological age could also represent an important prognostic factor only in younger subjects because of a ceiling effect in the relationship between severe COVID-19 and mortality in subjects older than 75. In fact, mortality was low under the age of 65 and peaked after the age of 75. Every small increment of age in the range 65-75 was probably associated with a substantial increase in the risk of death, while every small increment of age after 75 years old was not associated with further increases in the risk of death because mortality was already maximal in this age range.

Some limitations should be considered when interpreting the findings of our study. First, the retrospective design prevents to draw any robust causal inference on the role of PCT as predictor of adverse outcomes in COVID-19, but simply suggests an association requiring further confirmation in future studies with prospective design. The inclusion of patients who tested negative for SARS-CoV-2 on the day of admission, despite anamnestic, clinical, and radiological findings compatible with COVID-19, should be also considered as a potential source of bias, since the diagnosis was not confirmed in conformity with international standards. However, false-negative nasopharyngeal tests for SARS-CoV-2 were frequently reported during the early phases of the pandemic [25]. Individual susceptibility factors to severe clinical course, including genetic polymorphisms that have a known relationship with COVID-19-related mortality [51, 52], were not investigated.

Moreover, the particular circumstances of massive hospital overcrowding during the first month of the pandemic outbreak should be considered as another issue limiting the validity and generalizability of results. The relatively low rate of ICU admission should thus be interpreted in the context of an emergency situation with resource constraints. Information on long-term outcomes of survivors, after hospital discharge, were also unavailable. Such information could have been useful to verify whether hyperinflammatory state with PCT elevation on admission was associated with COVID-19 sequelae or late mortality. Finally, a thorough assessment of the possible presence of bacterial superinfections was not possible in a large number of patients.

Such limitations are however counterbalanced by remarkable points of strength. This is one of the few studies specifically focused on PCT elevation in oldest old patients. The large sample size of COVID-19 patients of different ages allowed us to verify the clinical significance of PCT elevation in both adult and geriatric patients, especially in relation to the burden of frailty and multimorbidity.

## 5. Conclusions

In a large group of patients hospitalized for suspect COVID-19 during the first pandemic wave in Italy, PCT elevation on admission was associated with other clinical, laboratory, and radiological parameters of disease severity. However, PCT elevation was significantly associated with hospital mortality risk only in subjects older than 75, suggesting that PCT may represent an excellent prognostic biomarker of COVID-19 especially in geriatric patients.

## Figures and Tables

**Table 1:** Overview of the main demographical, radiological, and virological characteristics on admission of the 1074 patients included in the study, stratified according to procalcitonin levels on admission.

	PCT < 0.05 ng/ml (*N* = 130)	PCT ≥0.05-<0.5 ng/ml (*N* = 698)	PCT ≥0.5-<2 ng/ml (*N* = 145)	PCT ≥ 2 ng/ml (*N* = 101)	*P*	*P* for trend
Age, years	65 (58-80)	74 (63-83)	78 (68-84)	78 (70-85)	**<0.001**	**<0.001**
Females, %	65	43	35	34	**<0.001**	**<0.001**
Consolidations on HRCT, %	56	70	65	73	**0.009**	0.069
HRCT visual score	20 (15-30)	30 (20-48)	40 (25-60)	35 (20-56)	**<0.001**	**<0.001**
First swab positive for SARS-CoV-2, %	55	65	69	69	0.082	**0.032**

Data reported as median and interquartile range (IQR) or percentage. *P* values calculated with Kruskal-Wallis or chi-square tests, *P* for trend with ANOVA. *P* values < 0.05 are indicated in bold (PCT: procalcitonin; HRCT: high-resolution computed tomography; SARS-CoV-2: Severe Acute Respiratory Syndrome CoronaVirus-2).

**Table 2:** Anamnestic and electrocardiographic characteristics the 1074 study participants, stratified according to procalcitonin levels on admission.

	PCT < 0.05 ng/ml (*N* = 130)	PCT ≥0.05-<0.5 ng/ml (*N* = 698)	PCT ≥0.5-<2 ng/ml (*N* = 145)	PCT ≥ 2 ng/ml (*N* = 101)	*P* ^∗^	*P* < 0.05 Standardized *β* or odds ratio
Personal history						
Total dependency in daily activities, %	9	18	15	30	**0.017**	1.33 (1.05-1.68)
Chronic diseases, number	2 (1-4)	3 (1-4)	3 (2-4)	4 (2-5)	**0.028**	0.062
Drugs, number	3 (0-6)	3 (1-6)	4 (2-7)	5 (3-8)	**0.002**	0.092
Hypertension, %	47	58	65	66	0.080	
Diabetes, %	14	19	24	27	0.225	
Obesity, %	8	12	16	12	**0.031**	1.31 (1.03-1.68)
Chronic heart disease, %	22	22	33	35	0.166	
Chronic kidney disease, %	2	5	10	20	**<0.001**	1.97 (1.48-2.62)
ACE-I treatment, %	16	24	26	22	0.946	
ARB treatment, %	20	16	18	23	0.723	
COVID-19-related symptoms						
Duration of symptoms, days	7 (4-10)	7 (4-10)	7 (4-10)	5 (3-7)	**0.034**	-0.069
Cough, %	50	48	47	32	0.065	
Dyspnea, %	45	52	61	64	**0.009**	1.26 (1.06-1.49)
Diarrhea, %	9	7	8	9	0.360	
Fever, %	75	84	80	85	0.124	
Electrocardiogram abnormalities on admission						
Electrocardiogram abnormalities, %	38	47	59	69	**<0.001**	1.44 (1.20-1.72)
Repolarization abnormalities, %	22	26	31	42	**0.001**	1.35 (1.12-1.62)
Atrial fibrillation, %	5	10	15	19	**0.008**	1.42 (1.10-1.84)
Other tachiarrythmias, %	2	4	6	13	**0.007**	1.59 (1.14-2.21)
QTC, msec	442 (420-461)	445 (427-467)	449 (437-480)	459 (436-487)	**0.019**	0.085

^∗^ *P* adjusted for age, sex, consolidations, and positive nasopharyngeal swab. Data shown as median and interquartile range (IQR) or percentage. *P* values calculated with linear regression for continuous variables and logistic regression for dichotomous variables (PCT: procalcitonin; COVID-19: CoronaVirus Disease-19; ACE-I: angiotensin-converting enzyme inhibitors; ARB: angiotensin receptor blocker; QTC: QT interval corrected according to the Bazin formula).

**Table 3:** Laboratory tests on admission of the 1074 study participants, stratified according to procalcitonin levels.

	PCT < 0.05 ng/ml (*N* = 130) (1)	PCT ≥0.05-<0.5 ng/ml (*N* = 698) (2)	PCT ≥0.5-<2 ng/ml (*N* = 145) (3)	PCT ≥ 2 ng/ml (*N* = 101) (4)	*P* ^∗^	*P* < 0.05 Bonferroni test
Blood gas analysis						
pH	7.44 (7.41-7.46)	7.45 (7.43-7.48)	7.44 (7.41-7.47)	7.42 (7.38-7.47)	**<0.001**	(4) vs. (2)
Bicarbonate, mmol/l	25 (24-27)	25 (23-27)	25 (22-28)	22 (20-25)	**<0.001**	(4) vs. (1) vs. (2) vs. (3)
PaCO_2_, mmHg	37 (35-41)	36 (33-39)	36 (32-40)	34 (30-38)	**0.005**	(4) vs. (1) vs. (2)
PaO_2_/FiO_2_, mmHg	357 (290-406)	257 (173-333)	141 (84-260)	171 (87-278)	**<0.001**	(1) vs. (2) vs. (3) vs. (4) (2) vs. (3)
Blood cell count						
Hemoglobin, g/dl	13.2 (12.2-14.2)	13.5 (12.2-14.6)	13.5 (11.8-14.6)	13.1 (10.6-14.4)	**0.001**	(4) vs. (1) vs. (2) vs. (3)
Platelets, 1000/mm^3^	219 (180-270)	209 (163-273)	209 (168-271)	197 (145-253)	0.157	
Neutrophils, n/mm^3^	3657 (2404-5189)	4981 (3540-7014)	7217 (4833-9829)	9007 (4726-12398)	**<0.001**	(1) vs. (2) vs. (3) vs. (4) (2) vs. (3) vs. (4)
Lymphocytes, n/mm^3^	1154 (856-1650)	914 (649-1247)	894 (560-1245)	630 (398-898)	**<0.001**	(1) vs. (2) vs. (3) vs. (4) (2) and (3) vs. (4)
Clinical chemistry and inflammatory indexes						
Creatinine, mg/dl	0.7 (0.6-0.9)	0.9 (0.7-1.1)	1.0 (0.9-1.6)	1.5 (0.9-2.7)	**<0.001**	(1) and (2) vs. (3) vs. (4); (3) vs. (4)
Urea, mg/dl	32 (24-41)	43 (31-59)	65 (41-98)	78 (55-127)	**<0.001**	(1) vs. (2) vs. (3) vs. (4); (2) vs. (3) vs. (4); (3) vs. (4)
Sodium, mEq/l	139 (137-140)	138 (135-140)	137 (135-140)	137 (134-140)	0.429	
Potassium, mEq/l	3.9 (3.6-4.2)	4.0 (3.7-4.3)	4.1 (3.8-4.5)	4.0 (3.7-4.6)	**0.014**	(2) vs. (4)
Total bilirubine, mg/dl	0.6 (0.5-0.7)	0.7 (0.5-0.9)	0.8 (0.6-1.0)	0.7 (0.5-0.9)	**0.023**	(1) vs. (3)
AST, IU/l	32 (25-38)	43 (31-65)	65 (42-95)	55 (32-99)	**<0.001**	(1) vs. (3) vs. (4); (2) vs. (3)
LDH, IU/l	257 (209-305)	340 (269-432)	440 (350-612)	399 (283-624)	**<0.001**	(1) vs. (2) vs. (3) vs. (4) (2) vs. (3) vs. (4)
CPK, IU/l	86 (54-155)	125 (65-251)	207 (104-551)	235 (113-802)	**<0.001**	(1) and (2) vs. (3) vs. (4)
Fibrinogen, ng/dl	502 (394-592)	596 (502-730)	708 (552-806)	648 (535-806)	**<0.001**	(1) vs. (2) vs. (3) vs. (4) (2) vs. (3)
CRP, mg/l	25 (5-55)	92 (50-143)	167 (111-230)	172 (111-250)	**<0.001**	(1) vs. (2) vs. (3) vs. (4) (2) vs. (3) vs. (4)
IL-6, pg/ml	31 (19-84)	126 (61-246)	173 (100-272)	236 (124-731)	**<0.001**	(4) vs. (1) vs. (2) vs. (3)
D-dimer, ng/dl	658 (464-1068)	990 (626-1740)	1374 (862-2392)	1514 (954-2679)	**<0.001**	(3) vs. (1) vs. (2)

^∗^ *P* calculated with univariate general linear model (GLM) and adjusted for age, sex, consolidations, and positive nasopharyngeal swab.

Data shown as median and interquartile range (IQR) (PCT: procalcitonin; PaCO_2_: arterial pressure of carbon dioxide; PaO_2_: arterial pressure of oxygen; FiO_2_: fraction of inspired oxygen; AST: aspartate aminotransferase; CPK: creatine phosphokinase; LDH: lactate dehydrogenase; CRP: C-reactive protein; IL-6: interleukin-6).

**Table 4:** Prevalence of the main laboratory abnormalities and outcomes of the 1074 study participants, stratified according to procalcitonin levels.

	PCT < 0.05 ng/ml (*N* = 130)	PCT ≥0.05-<0.5 ng/ml (*N* = 698)	PCT ≥0.5-<2 ng/ml (*N* = 145)	PCT ≥ 2 ng/ml (*N* = 101)	*P* ^∗^	*P* < 0.05 OR (95% CI)
Prevalence of laboratory abnormalities						
Neutrophil count > 8000/mm^3^, %	7	18	41	57	**<0.001**	2.65 (2.17-3.24)
Lymphocyte count < 1000/mm^3^, %	40	57	57	81	**<0.001**	1.48 (1.24-1.77)
Monocyte count < 200/mm^3^, %	6	10	17	25	**<0.001**	1.44 (1.42-2.28)
AST > 40 IU/l, %	31	51	69	63	**<0.001**	1.71 (1.42-2.07)
LDH > 247 IU/l, %	58	84	90	84	**<0.001**	1.69 (1.31-2.19)
CPK > 200 IU/l, %	25	38	62	63	**<0.001**	1.80 (1.50-2.16)
D − dimer > 500 ng/dl, %	69	87	94	94	**<0.001**	1.92 (1.38-2.67)
Fibrinogen > 400 ng/dl, %	73	89	91	88	**0.028**	1.38 (1.04-1.84)
Outcomes						
Noninvasive ventilation, %	4	10	17	10	**0.001**	1.55 (1.20-2.00)
ICU admission, %	1	4	9	3	**0.011**	1.65 (1.12-2.41)
Death, %	4	21	39	52		
Death (patients ≤ 75 years old), %	3	10	17	25		
Death (patients > 75 years old), %	5	33	55	68		

^∗^ *P* calculated with logistic regression and adjusted for age, sex, consolidations, and positive nasopharyngeal swab. Data expressed as percentages (PCT: procalcitonin; OR: odds ratio; CI: confidence interval; AST: aspartate aminotransferase; LDH: lactate dehydrogenase; CPK: creatine phosphokinase; ICU: intensive care unit).

**Table 5:** Multivariate Cox regression model, testing variables associated with hospital mortality.

	Hazard ratio	95% confidence interval	*P* ^a^
Model 1—patients aged 75 or younger (n = 550)			
Age, years	1.134	1.082-1.189	**<0.001**
Sex, female vs. male	0.993	0.558-1.768	0.981
ACE-I treatment	0.706	0.376-1.327	0.279
ARB treatment	1.144	0.588-2.225	0.692
Consolidations on chest HRCT	1.312	0.718-2.396	0.377
Nasopharyngeal swab positive for SARS-CoV-2	1.680	0.863-3.271	0.127
Procalcitonin^∗^			0.060
Level 2 vs. level 1	1.185	0.357-3.930	0.782
Level 3 vs. level 1	1.600	0.429-5.974	0.484
Level 4 vs. level 1	3.308	0.855-12.804	0.083
Model 2—patients aged > 75 years old (n = 524)			
Age, years	1.013	0.986-1.041	0.348
Sex, female vs. male	0.916	0.677-1.239	0.568
ACE-I treatment	1.016	0.714-1.446	0.929
ARB treatment	0.804	0.529-1.221	0.306
Consolidations on chest HRCT	1.130	0.817-1.564	0.459
Nasopharyngeal swab positive for SARS-CoV-2	1.816	1.268-2.602	**0.001**
Procalcitonin^∗^			**<0.001**
Level 2 vs. level 1	13.388	1.861-96.301	**0.010**
Level 3 vs. level 1	31.090	4.240-227.985	**0.001**
Level 4 vs. level 1	30.629	4.176-224.645	**0.001**

^∗^Procalcitonin level 1: <0.05 ng/ml; level 2: ≥0.05 and <0.5 ng/ml; level 3: ≥0.5 and <2 ng/ml; level 4: ≥2 ng/ml. ACE-I: angiotensin-converting enzyme inhibitors; ARB: angiotensin receptor blockers; HRCT: high-resolution computed tomography.

## Data Availability

Data are available from the authors, in rigorously anonymous form, upon reasonable request.
